# Relationship between serum iron and blood eosinophil counts in asthmatic adults: data from NHANES 2011-2018

**DOI:** 10.3389/fimmu.2023.1201160

**Published:** 2023-09-04

**Authors:** Jun Wen, Changfen Wang, Jing Xia, Mohan Giri, Shuliang Guo

**Affiliations:** ^1^ Department of Respiratory and Critical Care Medicine, The First Affiliated Hospital of Chongqing Medical University, Chongqing Medical University, Chongqing, China; ^2^ Department of Cardiology, The First Affiliated Hospital of Chongqing Medical University, Chongqing Medical University, Chongqing, China; ^3^ Department of Respiratory and Critical Care Medicine, The Third Affiliated Hospital of Chongqing Medical University, Chongqing Medical University, Chongqing, China

**Keywords:** serum iron, eosinophil, asthma, National Health And Nutrition Examination Survey (NHANES), XGBoost, machine learning

## Abstract

**Background:**

So far, quite a few studies have revealed that systemic iron levels are related to asthmatic inflammatory reactions. And most studies have focused on the correlation between systemic iron levels and asthma, with inconsistent findings. Yet, few studies have investigated the connection between serum iron and blood eosinophil counts. Hence, we have explored the connection between serum iron and blood eosinophil counts in asthmatics by utilizing data from NHANES.

**Methods:**

A total of 2549 individuals were included in our study after screening NHANES participants from 2011 to 2018. The linear regression model and XGBoost model were used to discuss the potential connection. Linear or nonlinear association was further confirmed by the generalized additive model and the piecewise linear regression model. And we also performed stratified analyses to figure out specific populations.

**Results:**

In the multivariable linear regression models, we discovered that serum iron levels were inversely related to blood eosinophil counts in asthmatic adults. Simultaneously, we found that for every unit increase in serum iron (umol/L), blood eosinophil counts reduced by 1.41/uL in model 3, which adjusted for all variables excluding the analyzed variables. Furthermore, the XGBoost model of machine learning was applied to assess the relative importance of chosen variables, and it was determined that vitamin C intake, age, vitamin B12 intake, iron intake, and serum iron were the five most important variables on blood eosinophil counts. And the generalized additive model and piecewise linear regression model further verify this linear and inverse association.

**Conclusion:**

Our investigation discovered that the linear and inverse association of serum iron with blood eosinophil counts in asthmatic adults, indicating that serum iron might be related to changes in the immunological state of asthmatics. Our work offers some new thoughts for next research on asthma management and therapy. Ultimately, we hope that more individuals become aware of the role of iron in the onset, development, and treatment of asthma.

## 1 Introduction

Asthma is a chronic inflammatory respiratory disease characterized by episodes of airflow obstruction (1). Asthma prevalence has risen exponentially in recent decades, particularly in Western and industrialized countries (2, 3). In some countries, up to 15-20% of the general population has a diagnosis of asthma, which is extremely concerning due to the public health issues caused by increased asthma exacerbations, hospital admissions, school absences, and high medical costs (4, 5). According to the World Health Organization, 15 million disability-adjusted life years are lost each year, and 250,000 asthma deaths are reported, accounting for approximately 1% of the global disease burden with an estimated prevalence of 358 million cases (6). And, the World Health Organization predicts that the number of asthmatics will rise by another 100 million by 2025 (7).

Asthma is a complex inflammatory disease of the airways with numerous pathophysiological features (8). Type 2 (T2) inflammation is a crucial immune response in the pathobiology of asthma, leading to the categorization of asthma into T2-high and T2-low classifications (9–11). It has been determined that eosinophils are the most important inflammatory cells in T2 high asthma (12). They are crucial effector cells that contribute to the pathogenesis of asthma by inducing type 2 inflammation, the primary asthma trigger (13). Eosinophils have been demonstrated to boost type 2 immune responses by producing cytokines and chemokines such as IL-4, IL-5, IL-9, and IL-13 (14–16). Many studies have indicated a correlation between elevated blood eosinophil levels and acute asthma attacks and asthma severity (17–20). Furthermore Robert et al. observed that a high eosinophil count in the blood of asthmatic patients could be a risk factor for future asthma exacerbations (21). In addition, eosinophils are the primary target of asthma and play a crucial role in asthma treatment. The novel interleukin-5-targeting biologics, for instance, can significantly reduce circulating blood eosinophils, which is associated with fewer asthma exacerbations and improved asthma management (22, 23). Eosinophils have a crucial role in the onset, development, and treatment of asthma (24).

It is thought that dietary changes may have contributed to the development of asthma over the past few decades as a result of society’s ongoing evolution (25). A considerable number of studies have shown that dietary micronutrients (such as iron, magnesium, calcium, copper, zinc, selenium, vitamin A and vitamin D, and so on) are related to the pathogenesis of atopic diseases (26–30). One of the important trace elements, iron, is crucial for many biological processes, including the regulation of enzyme activity, oxygen transport, and immune function (31), which may be the mechanism affecting asthma (32). Increased systemic iron doses were reported to dramatically reduce airway eosinophilia and Th2 cytokines, block AHR, and improve allergic asthma symptoms in a number of animal models (33–35). Moreover, numerous clinical studies have demonstrated and the connection between the level of iron within the human body and the occurrence of asthma (36–42).

Quite a few studies to date have focused on the correlation between systemic iron levels and asthma. Yet, few research have examined the relationship between eosinophil counts and serum iron levels in asthmatics. Using National Health and Nutrition Examination Survey (NHANES) data, we investigated the connection between serum iron concentrations and blood eosinophil counts in American adults with asthma to gain insight into the role of serum iron in asthma.

## 2 Materials and methods

### 2.1 Data source

Every two years, the National Health and Nutrition Examination Survey (NHANES), sponsored by the Centers for Disease Control and Prevention, collected data on the health and nutritional status of the U.S. population. NHANES uses a complex, multistage probability design to sample the civilian, noninstitutionalized population residing in the 50 states and D.C. The NCHS Institutional Review Board authorized the NHANES database in conformity with the revised Helsinki Declaration. Before the data collecting procedures and exhaustive health tests, the informed consent forms were completed. Details of the design and content of NHANES are available on the NHANES website.

### 2.2 Study population

**Figure 1:**
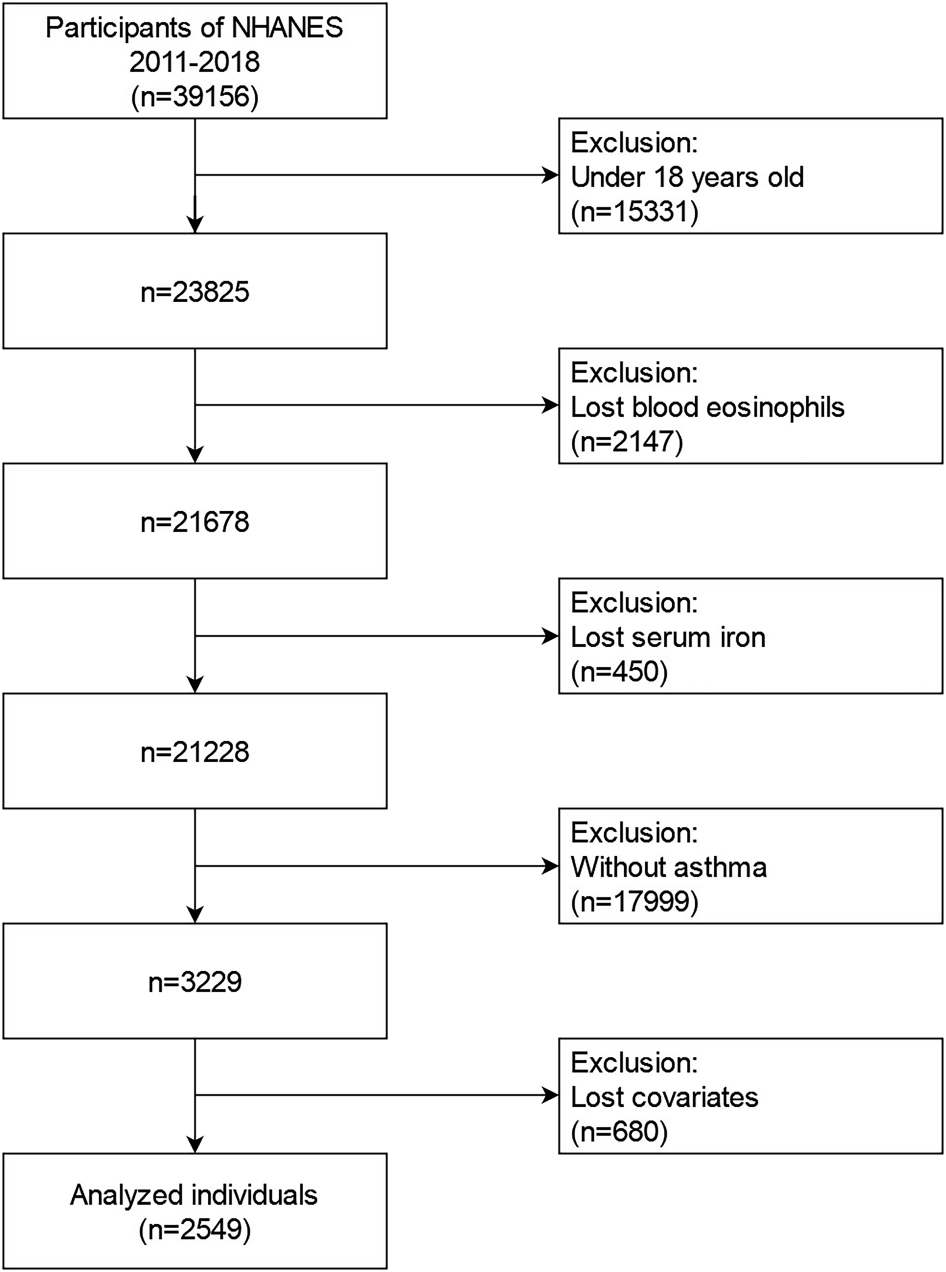
Flowchart for choosing asthmatic subjects.

The data from the 2011-2018 NHANES from were utilized in our investigation. For the second analyses, these data comprised demographic information, examination information, dietary information, laboratory information, and questionnaire data. During 2011 to 2018, the NHANES collected an aggregate of 39156 samples. We eliminated populations who were (1): younger than 18 years old (n=15331); (2) lost data on blood eosinophils (n=2147); (3) lost data on serum iron (n=450); (4) without asthma (n=17999); (5) lost data on more than one of following covariates(n=680): educational background, marital state, poverty to income ratios (PIR), body mass index (BMI), smoking state, alcohol intake, folate intake, vitamin A intake, vitamin B12 intake, vitamin C intake, iron intake, hypertension history, diabetes history, steroid use. Eventually, a large, nationally representative sample (n=2549) of asthmatic adults in the U.S. was recruited for our investigation. **Figure 1** depicts the flowchart for the screening procedure.

### 2.3 Measurement of serum iron and blood eosinophil counts

The serum iron concentration was measured using the DcX800 method, which is a timed-endpoint method. Acetic acid releases iron from transferrin, which is then reduced to the ferrous form by hydroxylamine and thioglycolate. The ferrous ion is complexed instantly with the FerroZine Iron Reagent. At a predetermined time period, the system measures the change in absorbance at 560 nm. This variation in absorbance is related to the iron concentration in the sample. Using a Becker Coulter MAXM analyzer, complete blood counts with 5-part differential measurements were done on whole blood samples obtained over the NHANES 2011–2018 cycles. The 5-part differential measure yielded cell counts of lymphocytes, monocytes, segmented neutrophils, eosinophils, and basophils (103 cells/L), which were utilized for *post-hoc* analyses. On the NHANES website, you can find a thorough overview of laboratory procedures.

### 2.4 Covariates and asthma assessment

Covariates comprised demographic information, examination information, dietary information, and questionnaire data. Demographic information involved sex, age (years), race (Mexican American, other Hispanic, non-Hispanic white, non-Hispanic black, others), educational background (less than high school, high school, more than high school), poverty to income ratios (grouped by trisection: low, middle, high; high poverty to income ratio means richer), marital state (married, single, living with a partner). Next, we also comprised examination information and diet information, such as body mass index (kg/m2), smoking state (whether smoked over 100 cigarettes in a lifetime), intake of alcohol, vitamin A, vitamin B12, vitamin C, folate and iron (average intake from two 24-hours recall data on diet and supplements), hypertension history (Yes, No), diabetes history (Yes, No, Borderline), and steroid drugs use history (whether steroid and other anti-allergy drugs use in past 30 days). The assessment of asthma was predicated on information obtained from the questionnaire segment of the National Health Interview Survey conducted in the United States. To evaluate the presence of asthma, the participants were queried with the following question: “Have you ever been diagnosed with asthma by a healthcare professional?” If the respondent answers affirmatively, they were classified as an individual with asthma. On the NHANES database website, you may find more comprehensive explanations of all variables.

### 2.5 Statistical analysis

We performed statistical analyses of serum iron concentrations and blood eosinophil counts in accordance with the NHANES database’s guidelines. Mean ± SD and percentage were used to illustrate continuous and categorical variables, individually. Initially, blood eosinophil counts were transformed into four quartiles. We calculated the p-value of categorical variables using the weighted chi-square test, and the p-value of continuous variables using the Kruskal Wallis rank sum test. The link of serum iron with blood eosinophil counts was then determined using three types of weighted linear regression models (Model 1, Model 2, Model 3). The model 1 adjusted no covariates; the model 2 adjusted sex, age, and race; and the model 3 adjusted gender, age, race, educational background, marital state, PIR, BMI, smoking state, alcohol intake, vitamin A intake, vitamin B12 intake, vitamin C intake, folate intake, iron intake, hypertension history, diabetes history, and steroid drug use. Next, we utilized the machine learning of XGBoost algorithm model to discuss the relative importance of selected variables on the effect of blood eosinophil counts. And, the stratified analyses were then undertaken to determine the stratified correlation between serum iron concentrations and blood eosinophil counts. To analyze the potential linear link of serum iron with blood eosinophil counts, we created a smooth curve according to the penalty spline approach using the generalized additive model. In addition, the segmented regression model was employed to confirm the linear or nonlinear association between serum iron concentrations and blood eosinophil counts. If a non-linear association was found, a two-piecewise linear regression model was used to assess the threshold influence of serum iron levels on blood eosinophil counts. When the ratio between serum iron levels and blood eosinophil counts became apparent in a smoothed curve, the recursive technique automatically estimates the inflection point at which the maximum model likelihood will be applied. R software (Version 4.2.0) with the R package was used to conduct all statistical analyses. In our research, a p-value of less than 0.05 indicated statistical significance.

## 3 Results

### 3.1 Baseline characteristics of analyzed individuals

Weighted distributions of the baseline characteristics, comprising demographic information, examination information, laboratory information, and questionnaire information from the 2011-2018 NHANES survey, were displayed in **Table 1**. In our investigation, the average age of selected participants was 47.3 years old, and non-Hispanic White people comprised the majority of the population. And afterwards, we quartiled the blood eosinophil counts (Q1–Q4). The distributions of sex, age, educational background, body mass index, smoking state, vitamin A intake, vitamin C intake, iron intake, hypertension history, and serum iron were statistically distinct (p value < 0.05), whereas the distributions of race, marital state, poverty to income ratio (PIR), alcohol intake, folate intake, vitamin B12 intake, diabetes history, and steroid drugs use were not statistically distinct (p value > 0.05). Relative to groups with the higher blood eosinophil count, groups with the lower blood eosinophil counts exhibited higher serum iron concentrations.

**Table 1:** Weighted characteristics of the study population in disaggregated by quartiles of blood eosinophil counts.

	Q1 (0)	Q2 (100)	Q3 (200)	Q4 (300-2200)	P value
Gender (%)					<0.0001
Male	34.01	32.11	38.59	45.06	
Female	65.99	67.89	61.41	54.94	
Age (years old)	45.93 ± 1.82	43.42 ± 0.74	46.33 ± 0.81	47.18 ± 0.71	0.0012
Race/ethnicity (%)					0.1971
Mexican American	4.99	5.09	5.4	6.43	
Other Hispanic	5.6	5.73	6.61	6.46	
Non-Hispanic White	62.91	68.85	67.58	68.38	
Non-Hispanic Black	22.6	12.21	11.48	10.76	
Other Race	3.91	8.12	8.93	7.97	
Education (%)					0.0033
Less than high school	13.7	10.8	14.26	14.12	
High school	16.21	18.05	19.69	24.15	
More than high school	70.09	71.15	66.05	61.73	
Marital status (%)					0.2471
Married	42.41	51.29	48.42	51.43	
Single	53.13	40.96	42.84	41.12	
Living with a partner	4.45	7.74	8.75	7.44	
Poverty to income ratio	2.51 ± 0.23	2.91 ± 0.09	2.74 ± 0.11	2.79 ± 0.1	0.1173
BMI (kg/m2)	29.32 ± 1.01	29.61 ± 0.37	30.86 ± 0.49	31.66 ± 0.42	0.0016
Smoked at least 100 cigarettes in life (%)					0.0004
Yes	50.77	43.75	45.25	53.49	
No	49.23	56.25	54.75	46.51	
Alcohol intake (gm)	14.62 ± 3.62	12.11 ± 1.4	12.23 ± 1.96	13.03 ± 1.76	0.8534
Folate intake (mcg)	367.6 ± 33.65	391.04 ± 13.11	419.59 ± 16.06	422.69 ± 16.15	0.1645
Vit A intake (mcg)	510.22 ± 56.72	620.82 ± 23.35	701.69 ± 26.69	639.68 ± 38.23	0.0238
Vit B12 intake (mcg)	4.62 ± 0.61	4.86 ± 0.16	5.37 ± 0.24	5.28 ± 0.27	0.1538
Vit C intake (mg)	56.54 ± 6.82	83.13 ± 5.38	78.52 ± 4.71	79.96 ± 4.31	0.027
Iron intake (mg)	12.88 ± 1.16	13.85 ± 0.29	14.61 ± 0.5	15.73 ± 0.6	0.0166
Hypertension (%)					0.0013
Yes	38.55	30.05	38.02	41.66	
No	61.45	69.95	61.98	58.34	
Diabetes (%)					0.0778
Yes	15.91	8.33	13.32	14.64	
No	80.94	88.72	84.37	82.74	
Borderline	3.15	2.95	2.31	2.61	
Steroid drugs use (%)					0.1466
Yes	20.74	14.2	16.12	19.08	
No	79.26	85.8	83.88	80.92	
Serum iron (umol/l)	15.53 ± 0.63	15.39 ± 0.31	14.14 ± 0.26	14.67 ± 0.28	0.0183

Data are presented as weighted means ± SD or proportions. Q1–Q4: Blood eosinophil counts have been grouped by quartile. Q1: Eosinophils number is 0 cells/uL; Q2: Eosinophils number is 100 cells/uL; Q3: Eosinophils number is 200 cells/uL; Q4: Eosinophils number is 300-2200 cells/uL. gm, gram; mg, milligram; mcg, microgram.

### 3.2 The relationships of serum iron concentrations and blood eosinophil counts

We utilized three weighted linear regression models to examine the relationship between serum iron and blood eosinophil levels in persons with asthma (**Table 2**). In accordance with the outcomes, we noticed a statistically significant inverse connection between serum iron levels and blood eosinophil counts in models 2 and 3, but not in model 1. Blood eosinophil counts fell by 1.57 (-2.74, -0.40)/ul for each increased unit of serum iron (umol/L) in model 2, which adjusted for gender, age, and race. Adjusted for sex, age, race, educational background, marital state, PIR, BMI, smoking state, alcohol intake, vitamin A intake, vitamin B12 intake, vitamin C intake, folate intake, iron intake, hypertension history, diabetes history, and steroid drugs use, model 3 revealed that blood eosinophil counts fell by 1.41 (-2.60, -0.23)/ul for each increased unit of serum iron (umol/L). And we observed the trend test was statistically significant in the model 2 (p for trend < 0.05), whereas not in the model 1 and 3 (p for trend > 0.05), which indicated serum iron was linearly associated with blood eosinophil counts in the model 2, but not in in the model 1 and 3. At the same time, we conducted linear regression analyses and trend testing on non-asthmatic populations who met the standards for inclusion and exclusion (**Supplementary Figure 1**). It was observed that serum iron exhibited a linear and inverse correlation with blood eosinophil counts in non-asthmatic populations (p < 0.05 and p for trend < 0.05) in **Supplementary Table 1**.

**Table 2:** Three weighted linear regression models explicate the link of the serum iron with blood eosinophils counts.

	Model 1	Model 2	Model 3
	β (95% CI) P value	β (95% CI) P value	β (95% CI) P value
Serum iron	-1.04 (-2.27, 0.18) 0.1008	-1.57 (-2.74, -0.40) 0.0111	-1.41 (-2.60, -0.23) 0.0256
Serum iron			
Q1 (1.8-10.19)	Reference	Reference	Reference
Q2 (10.20-13.59)	-5.17 (-33.32, 22.99) 0.7204	-13.34 (-41.31, 14.62) 0.3540	-11.63 (-39.83, 16.57) 0.4247
Q3 (13.60-17.69)	0.30 (-24.40, 25.00) 0.9813	-8.47 (-33.16, 16.21) 0.5041	-7.15 (-31.56, 17.26) 0.5698
Q4 (17.70-47.60)	-15.78 (-39.21, 7.66) 0.1921	-26.82 (-48.79, -4.85) 0.0204	-24.14 (-46.91, -1.37) 0.0456
P for trend	0.2312	0.0299	0.0561

Model 1 adjusted no covariates. Model 2 adjusted sex, age and race. Model 3 adjusted sex, age, race, educational background, marital state, poverty to income ratio, body mass index, smoking state, alcohol intake, vitamin A intake, vitamin B12 intake, vitamin C intake, folate intake, iron intake, hypertension history, diabetes history, and steroid drugs use. Q1-Q4: Serum iron are grouped by quartile.

### 3.3 Stratification connection of serum iron with blood eosinophil counts

**Figure 2:**
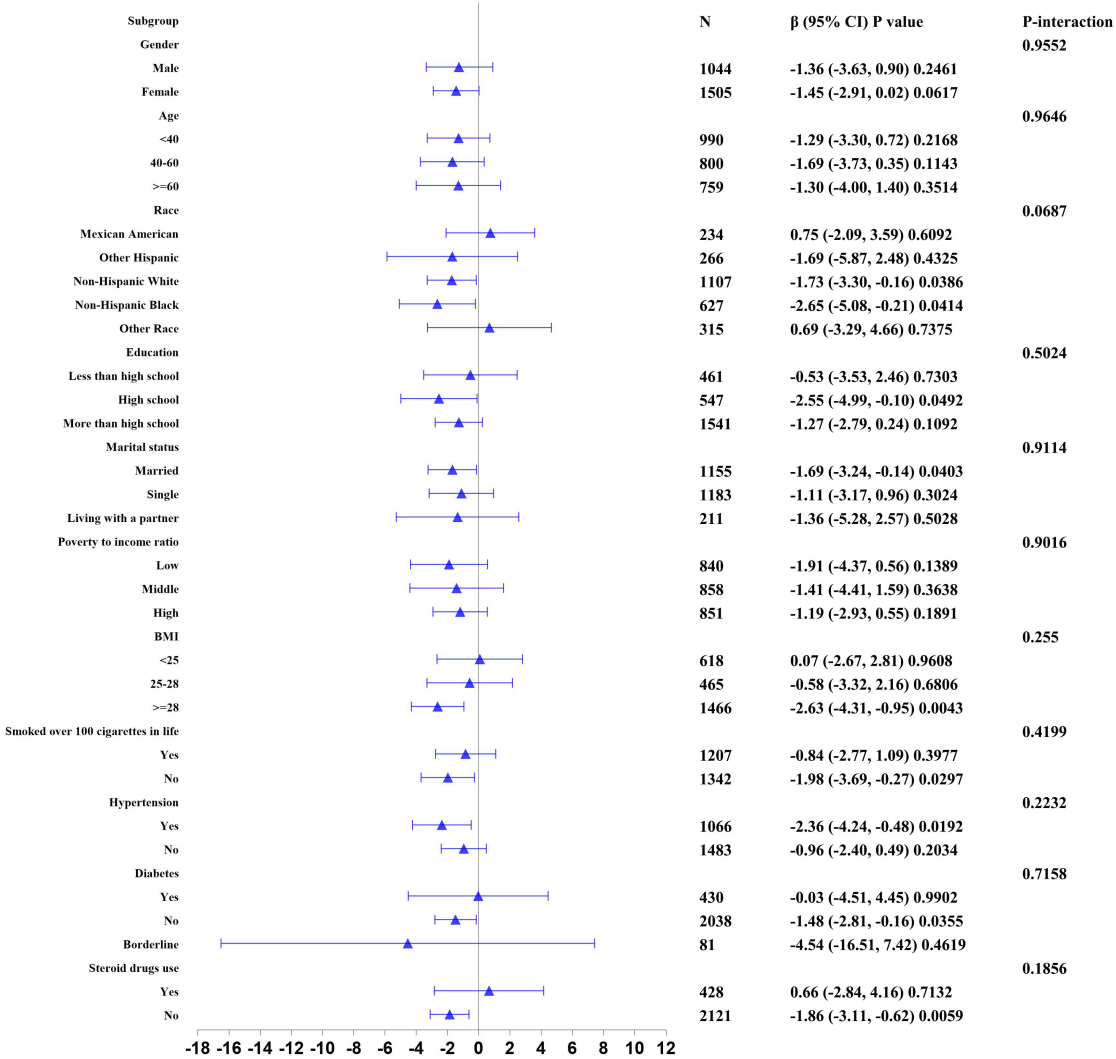
Stratified correlation of serum iron with blood eosinophil counts. In any of subgroups, the model adjusted sex, age, race, educational background, marital state, poverty to income ratio, body mass index, smoking state, alcohol intake, vitamin A intake, vitamin B12 intake, vitamin C intake, folate intake, iron intake, hypertension history, diabetes history, and steroid drugs use except for the stratification variable.

We further analyzed stratification connection of serum iron and blood eosinophil counts in different subgroups by sex, age, race, education background, marital state, PIR, BMI, smoking state, hypertension history, diabetes history, and steroid drugs use to ensure that the results of the linear regression analysis were reliable (**Figure 2**). According to stratified analysis results, we discovered that a negative connection of serum iron with blood eosinophil counts in the specific populations, who were Non-Hispanic White and Black individuals, high school, married state, BMI≥28, smoking over 100 cigarettes in life, with hypertension, without diabetes and without steroid drug use. In addition, the interaction test did not find any statistically significant differences among all subgroups. (p for interaction > 0.05). At the same time, we also conducted a stratified analysis of the non-asthmatic population. And we discovered a negative correlation of serum iron with blood eosinophil counts in other subgroup populations except for these populations, who were age over 40, other Hispanic individuals, high school and below, low PIR, BMI below 25, with hypertension, borderline diabetes and diabetes, and with steroid drug use. Similarly, no interaction was found in all subgroup analyses (p for interaction > 0.05) in **Supplementary Table 2**.

### 3.4 Assessing the relative importance of selected variables by the XGBoost algorithm model

**Figure 3:**
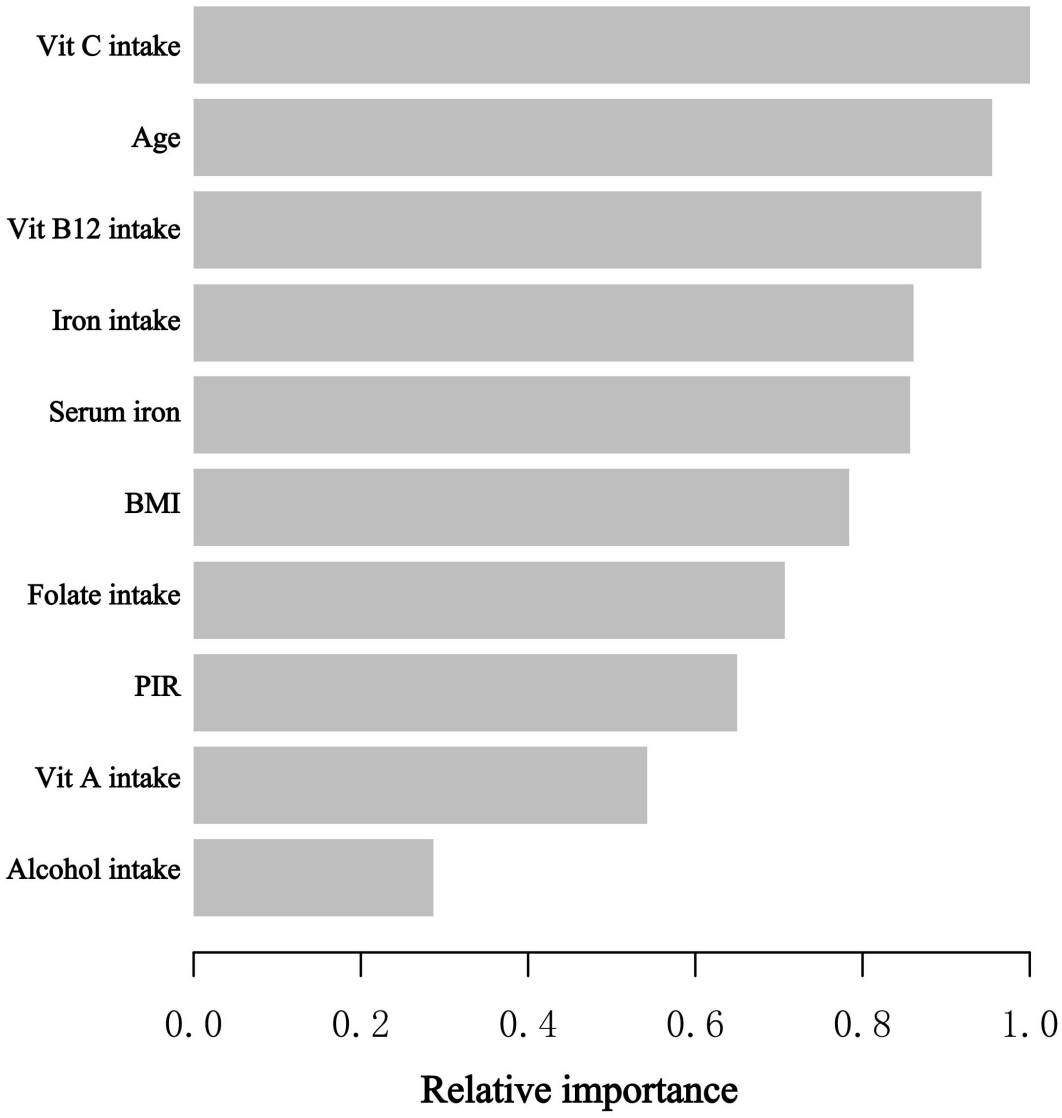
The XGBoost algorithm determines the relative importance of each variable on blood eosinophil counts and assigns a variable importance score to each variable. The X-axis represents the importance score, which is the relative importance of variables used to distribute the data; the Y-axis represents the variables chosen. PIR, poverty to income ratio; Vit, Vitamin.

In the stages of model development and verification, we implemented the XGBoost algorithmic model of machine learning to assess the relative significance of selected variables associated with blood eosinophil counts. The selected variables consisted of age, poverty-to-income ratio, body mass index, alcohol consumption, intake of vitamin A, vitamin B12, vitamin C, folate, iron, and serum iron. On the basis of the outcomes of each variable’s contribution by XGBoost model, we discovered that vitamin C intake, age, vit B12 intake, iron intake, and serum iron were the five most influential factors in the blood eosinophil counts (**Figure 3**). Serum iron, as a relatively crucial variable, was subsequently incorporated into the general additive model and segmented regression model in order to further evaluate the reliability of the linear regression analysis results in our investigation.

### 3.5 Evaluating the linear or nonlinear associations of serum iron levels and blood eosinophil counts

**Figure 4:**
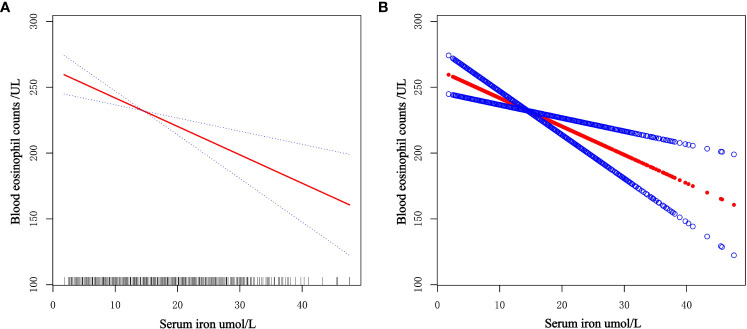
Dose-response connection of serum iron levels with blood eosinophil counts based on model 3. **(A)** The red solid line is the smooth fitting curve between serum iron levels and blood eosinophil counts, while the blue dashed line reflects the 95% confidence intervals of the fitting. **(B)** Each red dot represents a separate sample, and the blue dots above and below the red dot represent 95% confidence intervals.

The general additive model (GAM) is extremely sensitive to determining whether a correlation is linear or nonlinear. We performed GAM to explore the linear or nonlinear connection of serum iron and blood eosinophil counts to validate the trustworthiness of regression analysis results. We generated a smooth fit curve based on model 3 (**Figure 4**) to illustrate the potential connection. We observed the linear connection of serum iron concentrations with blood eosinophil counts in adults with asthma after controlling all variables apart from serum iron. Furthermore, we implemented the segmented regression model to confirm the linearity or nonlinearity of the connection between serum iron and blood eosinophil counts (**Table 3**). Our study revealed that the inflection point (K=8.2) lacked statistical significance, as indicated by a log-likelihood ratio greater than 0.05. Additionally, there were no statistically significant distinctions observed between the one-line model and the segmented regression model. Thus, it can be concluded that the one-line model was a more suitable approach for elucidating the correlation of serum iron with blood eosinophil counts. All of above outcomes suggested a linear and inverse relationship of serum iron and blood eosinophil counts.

**Table 3:** Analysis of the threshold effect of serum iron and blood eosinophil counts implementing the two-piecewise linear regression model.

	β (95% CI) P value
Model 1 (one-line model)	
linear effect	-1.41 (-2.56, -0.27) 0.0158
Model 2 (two-piecewise linear regression model)	
Inflection point (K)	8.2
Serum iron< K	-7.98 (-16.89, 0.92) 0.0790
Serum iron> K	-0.99 (-2.27, 0.29) 0.1305
Log likelihood ratio	0.143

The model 1 and 2 all adjusted sex, age, race, educational background, marital state, poverty to income ratio, body mass index, smoking state, alcohol intake, vitamin A intake, vitamin B12 intake, vitamin C intake, folate intake, iron intake, hypertension history, diabetes history, and steroid drugs use.

## 4 Discussion

In recent years, there has been a significant increase in allergic illnesses, which has accompanied with a remarkable increase in interest in the potential link between micronutrients and asthma (26, 31, 43). Iron is by far one of the most abundant and significant transition metals in nature, serving critical biological activities in a variety of biological processes including DNA and RNA synthesis, enzyme activity, inflammation, and oxidative stress (44). Iron may influence the likelihood of asthma attacks or exacerbations via affecting biological processes. Previous studies have shown that asthmatic chronic inflammatory reactions are related to lower systemic iron levels (45, 46).

As a result, we evaluated the relationship between serum iron levels and blood eosinophil counts in 2,549 adults with asthma who took part in the NHANES survey in the United States from 2011 to 2018. To our knowledge, our investigation is the first study to discuss the connection between serum iron levels and blood eosinophil counts in adults with asthma and one of the most extensive cross-sectional studies. In multiple linear regression models, we observed the negative correlation between serum iron levels and blood eosinophil counts in American adults with asthma. Simultaneously, we discovered that for every unit increase in serum iron (umol/L), blood eosinophil counts reduced by 1.41/uL in model 3, which controlled for sex, age, race, educational background, marital state, PIR, BMI, smoked state, alcohol intake, vitamin A intake, vitamin B12 intake, vitamin C intake, folate intake, iron intake, hypertension history, diabetes history, and steroid drug use. Furthermore, we created a machine learning XGBoost model to estimate the relative importance of selected variables, and found that vitamin C intake, age, vit B12 intake, iron intake, and serum iron were the five most influential factors on blood eosinophil counts. Then, we determined the linear and negative association between serum iron levels and blood eosinophil counts by the generalized additive model and the two-piecewise linear regression model. As shown by the findings discussed above, the correlation between serum iron and blood eosinophil count in American adults with asthma was linear and negative.

The majority of asthma cases are caused by type 2 inflammation, which is mediated by respiratory epithelium and type 2 T-helper lymphocytes. Inflammation of the airway is most likely caused by a combination of changes in geographical location, environmental factors, and dietary patterns (47, 48). It is associated with certain cytokine profiles (IL-4, IL-5, and IL-14) and inflammatory cells (eosinophils, basophils, type 2 T helper lymphocytes, and IgE-producing plasma cells) (49). Inflammation obstructs the bronchial airways, producing wheezing and coughing as clinical symptoms, which triggers asthma attacks. As a vital dietary trace element, iron is important for cellular respiration and electron transport, oxygen metabolism, DNA synthesis, gene control, drug metabolism, and steroid synthesis (44). Deficits in essential biological processes brought on by an iron deficiency might have negative effects. And it has been proved that iron is involved in controlling inflammatory reactions in asthma (50, 51).

Quite a few published studies reported the connection between asthma and iron intake. A case-control study found that iron supplementation improved chronic cough and bronchial hyperreactivity (52). Haim Bibi et al. found that iron-chelating complex attenuated allergic airway inflammation in this mouse model of allergic asthma (53). Moreover, another study indicated that in a mouse model of IgE-mediated allergic asthma, iron supplementation could beneficially decrease the severity of allergic asthma (54). The above studies indicated that sufficient iron intake levels may contribute to a protective effect on asthma. Maazi et al. observed that the administration of iron supplements causes a noteworthy reduction in airway eosinophilia. Additionally, the use of systemic iron injections resulted in a significant suppression of both allergen-induced airway eosinophilia and hyperreactivity in comparison to the administration of a placebo. But compared with their iron-sufficient counterparts, mice fed on an iron-deprived diet did not exhibit any discernible variance in the development of experimentally induced allergic asthma (28). However, the above studies did not indicate whether there was information about iron absorption disorders, so the conclusions were not completely consistent. In addition, some researches have investigated the association between iron status and asthma. For instance, according to a study conducted in the United States, a higher ferritin level was associated with a decreased risk of lifetime asthma, current asthma, and asthma attacks in women (55). Another case-control study involving 1102 matched participants revealed a negative association between asthma prevalence and urine iron levels in Chinese adults (56). Other researchers, however, obtained different results. For instance, Narula MK et al. observed that elevated plasma iron levels in asthmatics may lead to worsening lipid peroxidation, indicating a link between plasma iron levels and asthma severity (57). And a Japanese study comprising 1 025 patients revealed that serum iron and bronchial asthma were not associated (58). A variety of confounders might well have led to inconsistent findings in previous studies. Yet, our study revealed a negative correlation between serum iron and blood eosinophil count in American adults with asthma, indicating that serum iron was associated with changes in the immunological state of asthmatics.

Compared to past studies, ours has some advantages. Our study presents a rather large, nationally representative sample of individuals with asthma and involves quite a few potential confounders. Second, we use stratified analysis to determine the connection between serum iron and blood eosinophil counts in the different population because confounders may affect the results. Then, we use the XGBoost algorithm model’s machine learning to determine the relative importance of selected variables. And the linear and inverse relationship between serum iron and blood eosinophil counts is further confirmed by generalized additive model and segmented regression model. Our work offers some new thoughts for next research on asthma management and therapy. More studies will be required in the future to clarify the connection between serum iron and blood eosinophil counts and to determine the underlying mechanism.

Despite this, we recognize that our study has some limitations. Due to the limitations of the NHANES database, we included asthmatic individuals based on questionnaire data as opposed to the lung function test. And the pharmaceuticals included in our study that altered blood eosinophils were predominantly cortisol drugs and other anti-allergy medications, but no biologics. Owing to constraints in the NHANES database, our analysis includes only serum iron but not iron binding capability, transferrin saturation, and information of pre and peri-birth nutrition. The XGBoost algorithm remains an algorithm, created by humans, and as such is not guaranteed to be 100% objective. And participants’ physical conditions at the time of blood collection, such as whether they have acute or stable asthma, are not known. Therefore, additional prospective studies will be necessary to shed light on the potential role of serum iron in the control, progression, and treatment of asthma and to uncover potential mechanisms of action.

## 5 Conclusion

Our investigation discovered that the linear and inverse association between serum iron and blood eosinophil counts in asthmatic adults, indicating that serum iron might be related to changes in the immunological state of asthmatics. Our work offers some new thoughts for next research on asthma management and therapy. Ultimately, we look forward to more people recognizing the role of iron in the onset, development, and treatment of asthma.

## Data availability statement

Publicly available datasets were analyzed in this study. This data can be found here: http://www.cdc.gov/nchs/nhanes/.

## Ethics statement

The studies involving humans were approved by NCHS Research Ethics Review Board. The studies were conducted in accordance with the local legislation and institutional requirements. The human samples used in this study were acquired from The NHANES protocol was approved by the NCHS Research Ethics Review Board. Written informed consent for participation was not required from the participants or the participants’ legal guardians/next of kin in accordance with the national legislation and institutional requirements.

## Author contributions

JW performed the study design, data extraction, statistical analysis, drafted the manuscript, and revised the manuscript. CW performed the study design, data extraction, and drafting of the manuscript. JX re-collected, cleaned and analyzed the data, participated in the revision of the manuscript, and played an important role in the revision of the manuscript. MG performed data extraction and statistical analysis and revised the manuscript. SG participated in the study’s design and management and revised the manuscript. All authors read and approved the final manuscript.

## References

[1] Porsbjerg C Melén E Lehtimäki L Shaw D. Asthma. Lancet (London England) (2023) 401(10379):858–73. doi: 10.1016/S0140-6736(22)02125-0 36682372

[2] Reddel HK Bacharier LB Bateman ED Brightling CE Brusselle GG Buhl R. Global Initiative for Asthma Strategy 2021: executive summary and rationale for key changes. Eur Respir J (2022) 59(1):2102730. doi: 10.1183/13993003.02730-2021 34667060 PMC8719459

[3] Forno E Brandenburg DD Castro-Rodriguez JA Celis-Preciado CA Holguin F Licskai C. Asthma in the Americas: an update: A joint perspective from the Brazilian thoracic society, Canadian thoracic society, Latin American thoracic society, and american thoracic society. Ann Am Thorac Society (2022) 19(4):525–35. doi: 10.1513/AnnalsATS.202109-1068CME PMC8996271 35030062

[4] Bridevaux PO Probst-Hensch NM Schindler C Curjuric I Felber Dietrich D Braendli O. Prevalence of airflow obstruction in smokers and never-smokers in Switzerland. Eur Respir J (2010) 36(6):1259–69. doi: 10.1183/09031936.00004110 20413537

[5] Chipps BE Corren J Israel E Katial R Lang DM Panettieri RA Jr.. Asthma Yardstick: Practical recommendations for a sustained step-up in asthma therapy for poorly controlled asthma. Ann allergy Asthma Immunol (2017) 118(2):133–42.e3. doi: 10.1016/j.anai.2016.12.010 28153079

[6] Soriano JB Abajobir AA Abate KH Abera SF Agrawal A Ahmed MB. Global, regional, and national deaths, prevalence, disability-adjusted life years, and years lived with disability for chronic obstructive pulmonary disease and asthma, 1990-2015: a systematic analysis for the Global Burden of Disease Study 2015. Lancet Respir Med (2017) 5(9):691–706. doi: 10.1016/S2213-2600(17)30293-X 28822787 PMC5573769

[7] Asher MI García-Marcos L Pearce NE Strachan DP. Trends in worldwide asthma prevalence. Eur Respir J (2020) 56(6):2002094. doi: 10.1183/13993003.02094-2020 32972987

[8] Wen J Wang C Giri M Guo S. Association between serum folate levels and blood eosinophil counts in American adults with asthma: Results from NHANES 2011-2018. Front Immunol (2023) 14:1134621. doi: 10.3389/fimmu.2023.1134621 36911740 PMC9993087

[9] Kaur R Chupp G. Phenotypes and endotypes of adult asthma: Moving toward precision medicine. J Allergy Clin Immunol (2019) 144(1):1–12. doi: 10.1016/j.jaci.2019.05.031 31277742

[10] Maison N Omony J Illi S Thiele D Skevaki C Dittrich AM. T2-high asthma phenotypes across lifespan. Eur Respir J (2022) 60(3):2102288. doi: 10.1183/13993003.02288-2021 35210326 PMC9520028

[11] Roth-Walter F Adcock IM Benito-Villalvilla C Bianchini R Bjermer L Caramori G. Comparing biologicals and small molecule drug therapies for chronic respiratory diseases: An EAACI Taskforce on Immunopharmacology position paper. Allergy (2019) 74(3):432–48. doi: 10.1111/all.13642 30353939

[12] Schleich FN Chevremont A Paulus V Henket M Manise M Seidel L. Importance of concomitant local and systemic eosinophilia in uncontrolled asthma. Eur Respir J (2014) 44(1):97–108. doi: 10.1183/09031936.00201813 24525441

[13] James A Janson C Malinovschi A Holweg C Alving K Ono J. Serum periostin relates to type-2 inflammation and lung function in asthma: Data from the large population-based cohort Swedish GA(2)LEN. Allergy (2017) 72(11):1753–60. doi: 10.1111/all.13181 28398635

[14] Kubo M. Innate and adaptive type 2 immunity in lung allergic inflammation. Immunol Rev (2017) 278(1):162–72. doi: 10.1111/imr.12557 28658559

[15] Pagnoux C Nair P Xi Y Khalidi NA Carette S Cuthbertson D. Serum cytokine and chemokine levels in patients with eosinophilic granulomatosis with polyangiitis, hypereosinophilic syndrome, or eosinophilic asthma. Clin Exp Rheumatol (2019) 37 Suppl 117(2):40–4. PMC9878582 30652675

[16] Agache I Strasser DS Klenk A Agache C Farine H Ciobanu C. Serum IL-5 and IL-13 consistently serve as the best predictors for the blood eosinophilia phenotype in adult asthmatics. Allergy (2016) 71(8):1192–202. doi: 10.1111/all.12906 27060452

[17] Tran TN Khatry DB Ke X Ward CK Gossage D. High blood eosinophil count is associated with more frequent asthma attacks in asthma patients. Ann allergy Asthma Immunol (2014) 113(1):19–24. doi: 10.1016/j.anai.2014.04.011 24846699

[18] Katz LE Gleich GJ Hartley BF Yancey SW Ortega HG. Blood eosinophil count is a useful biomarker to identify patients with severe eosinophilic asthma. Ann Am Thorac Society (2014) 11(4):531–6. doi: 10.1513/AnnalsATS.201310-354OC 24606022

[19] Volbeda F Broekema M Lodewijk ME Hylkema MN Reddel HK Timens W. Clinical control of asthma associates with measures of airway inflammation. Thorax (2013) 68(1):19–24. doi: 10.1136/thoraxjnl-2012-201861 23042704

[20] Nadif R Siroux V Oryszczyn MP Ravault C Pison C Pin I. Heterogeneity of asthma according to blood inflammatory patterns. Thorax (2009) 64(5):374–80. doi: 10.1136/thx.2008.103069 19131450

[21] Zeiger RS Schatz M Li Q Chen W Khatry DB Gossage D. High blood eosinophil count is a risk factor for future asthma exacerbations in adult persistent asthma. J Allergy Clin Immunol In practice (2014) 2(6):741–50. doi: 10.1016/j.jaip.2014.06.005 25439366

[22] Jacobsen EA Jackson DJ Heffler E Mathur SK Bredenoord AJ Pavord ID. Eosinophil knockout humans: uncovering the role of eosinophils through eosinophil-directed biological therapies. Annu Rev Immunol (2021) 39:719–57. doi: 10.1146/annurev-immunol-093019-125918 PMC8317994 33646859

[23] FitzGerald JM Bleecker ER Nair P Korn S Ohta K Lommatzsch M. Benralizumab, an anti-interleukin-5 receptor α monoclonal antibody, as add-on treatment for patients with severe, uncontrolled, eosinophilic asthma (CALIMA): a randomised, double-blind, placebo-controlled phase 3 trial. Lancet (London England) (2016) 388(10056):2128–41. doi: 10.1016/S0140-6736(16)31322-8 27609406

[24] Wen J Giri M Xu L Guo S. Association between exposure to selected heavy metals and blood eosinophil counts in asthmatic adults: results from NHANES 2011-2018. J Clin Med (2023) 12(4):1543. doi: 10.3390/jcm12041543 36836077 PMC9965605

[25] Aydogan M Ozen A Akkoc T Eifan AO Aktas E Deniz G. Risk factors for persistence of asthma in children: 10-year follow-up. J Asthma (2013) 50(9):938–44. doi: 10.3109/02770903.2013.831872 23919566

[26] Alwarith J Kahleova H Crosby L Brooks A Brandon L Levin SM. The role of nutrition in asthma prevention and treatment. Nutr Rev (2020) 78(11):928–38. doi: 10.1093/nutrit/nuaa005 PMC7550896 32167552

[27] Peroni DG Hufnagl K Comberiati P Roth-Walter F. Lack of iron, zinc, and vitamins as a contributor to the etiology of atopic diseases. Front Nutr (2022) 9:1032481. doi: 10.3389/fnut.2022.1032481 36698466 PMC9869175

[28] Drury KE Schaeffer M Silverberg JI. Association between atopic disease and anemia in US children. JAMA pediatrics (2016) 170(1):29–34. doi: 10.1001/jamapediatrics.2015.3065 26619045

[29] Petje LM Jensen SA Szikora S Sulzbacher M Bartosik T Pjevac P. Functional iron-deficiency in women with allergic rhinitis is associated with symptoms after nasal provocation and lack of iron-sequestering microbes. Allergy (2021) 76(9):2882–6. doi: 10.1111/all.14960 PMC8453563 34037999

[30] Roth-Walter F. Iron-deficiency in atopic diseases: innate immune priming by allergens and siderophores. Front Allergy (2022) 3:859922. doi: 10.3389/falgy.2022.859922 35769558 PMC9234869

[31] Rouault TA Tong WH. Iron-sulfur cluster biogenesis and human disease. Trends Genet (2008) 24(8):398–407. doi: 10.1016/j.tig.2008.05.008 18606475 PMC2574672

[32] Rubin RN Navon L Cassano PA. Relationship of serum antioxidants to asthma prevalence in youth. Am J Respir Crit Care Med (2004) 169(3):393–8. doi: 10.1164/rccm.200301-055OC 14630617

[33] Groenman FA Rutter M Wang J Caniggia I Tibboel D Post M. Effect of chemical stabilizers of hypoxia-inducible factors on early lung development. Am J Physiol Lung Cell Mol Physiol (2007) 293(3):L557–67. doi: 10.1152/ajplung.00486.2006 17545484

[34] Maazi H Shirinbak S Bloksma N Nawijn MC van Oosterhout AJ. Iron administration reduces airway hyperreactivity and eosinophilia in a mouse model of allergic asthma. Clin Exp Immunol (2011) 166(1):80–6. doi: 10.1111/j.1365-2249.2011.04448.x PMC3193922 21910724

[35] Xu W Deng H Hu S Zhang Y Zheng L Liu M. Role of ferroptosis in lung diseases. J Inflammation Res (2021) 14:2079–90. doi: 10.2147/JIR.S307081 PMC8144020 34045882

[36] Huang L Li L Luo X Huang S Hou Q Ge X. The association between serum iron status and risk of asthma: a 2-sample Mendelian randomization study in descendants of Europeans. Am J Clin Nutr (2019) 110(4):959–68. doi: 10.1093/ajcn/nqz162 31380560

[37] Mao S Wu L Shi W. Association between trace elements levels and asthma susceptibility. Respir Med (2018) 145:110–9. doi: 10.1016/j.rmed.2018.10.028 30509699

[38] Chang JE Lee HM Kim J Rhew K. Prevalence of anemia in pediatric patients according to asthma control: propensity score analysis. J Asthma Allergy (2021) 14:743–51. doi: 10.2147/JAA.S318641 PMC8254559 34234469

[39] Rhew K Brown JD Oh JM. Atopic disease and anemia in korean patients: cross-sectional study with propensity score analysis. Int J Environ Res Public Health (2020) 17(6):1978. doi: 10.3390/ijerph17061978 32197291 PMC7142528

[40] Rhew K Oh JM. Association between atopic disease and anemia in pediatrics: a cross-sectional study. BMC pediatrics (2019) 19(1):455. doi: 10.1186/s12887-019-1836-5 31760939 PMC6876088

[41] Bédard A Lewis SJ Burgess S Henderson AJ Shaheen SO. Maternal iron status during pregnancy and respiratory and atopic outcomes in the offspring: a Mendelian randomisation study. BMJ Open Respir Res (2018) 5(1):e000275. doi: 10.1136/bmjresp-2018-000275 PMC5890059 29636978

[42] Shaheen SO Gissler M Devereux G Erkkola M Kinnunen TI McArdle H. Maternal iron supplementation in pregnancy and asthma in the offspring: follow-up of a randomised trial in Finland. Eur Respir J (2020) 55(6):1902335. doi: 10.1183/13993003.02335-2019 32139461

[43] Venter C Meyer RW Nwaru BI Roduit C Untersmayr E Adel-Patient K. EAACI position paper: Influence of dietary fatty acids on asthma, food allergy, and atopic dermatitis. Allergy (2019) 74(8):1429–44. doi: 10.1111/all.13764 31032983

[44] Pantopoulos K Porwal SK Tartakoff A Devireddy L. Mechanisms of mamMalian iron homeostasis. Biochemistry (2012) 51(29):5705–24. doi: 10.1021/bi300752r PMC3572738 22703180

[45] Kemna E Pickkers P Nemeth E van der Hoeven H Swinkels D. Time-course analysis of hepcidin, serum iron, and plasma cytokine levels in humans injected with LPS. Blood (2005) 106(5):1864–6. doi: 10.1182/blood-2005-03-1159 15886319

[46] Cherayil BJ. Pathophysiology of iron homeostasis during inflammatory states. J pediatrics (2015) 167(4 Suppl):S15–9. doi: 10.1016/j.jpeds.2015.07.015 PMC4568438 26364019

[47] Chung KF Wenzel SE Brozek JL Bush A Castro M Sterk PJ. International ERS/ATS guidelines on definition, evaluation and treatment of severe asthma. Eur Respir J (2014) 43(2):343–73. doi: 10.1183/09031936.00202013 24337046

[48] Custovic A Johnston SL Pavord I Gaga M Fabbri L Bel EH. EAACI position statement on asthma exacerbations and severe asthma. Allergy (2013) 68(12):1520–31. doi: 10.1111/all.12275 PMC7159478 24410781

[49] Fahy JV. Type 2 inflammation in asthma–present in most, absent in many. Nat Rev Immunol (2015) 15(1):57–65. doi: 10.1038/nri3786 25534623 PMC4390063

[50] Huang SK Zhang Q Qiu Z Chung KF. Mechanistic impact of outdoor air pollution on asthma and allergic diseases. J Thorac disease (2015) 7(1):23–33. doi: 10.3978/j.issn.2072-1439.2014.12.1 25694815 PMC4311071

[51] Ali MK Kim RY Karim R Mayall JR Martin KL Shahandeh A. Role of iron in the pathogenesis of respiratory disease. Int J Biochem Cell Biol (2017) 88:181–95. doi: 10.1016/j.biocel.2017.05.003 28495571

[52] Bucca C Culla B Brussino L Ricciardolo FL Cicolin A Heffler E. Effect of iron supplementation in women with chronic cough and iron deficiency. Int J Clin practice (2012) 66(11):1095–100. doi: 10.1111/ijcp.12001 23067033

[53] Bibi H Vinokur V Waisman D Elenberg Y Landesberg A Faingersh A. Zn/Ga-DFO iron-chelating complex attenuates the inflammatory process in a mouse model of asthma. Redox Biol (2014) 2:814–9. doi: 10.1016/j.redox.2014.06.009 PMC4085351 25009783

[54] Hale LP Kant EP Greer PK Foster WM. Iron supplementation decreases severity of allergic inflammation in murine lung. PLoS One (2012) 7(9):e45667. doi: 10.1371/journal.pone.0045667 23029172 PMC3447873

[55] Brigham EP McCormack MC Takemoto CM Matsui EC. Iron status is associated with asthma and lung function in US women. PLoS One (2015) 10(2):e0117545. doi: 10.1371/journal.pone.0117545 25689633 PMC4331366

[56] Huang X Xie J Cui X Zhou Y Wu X Lu W. Association between concentrations of metals in urine and adult asthma: A case-control study in Wuhan, China. PLoS One (2016) 11(5):e0155818. doi: 10.1371/journal.pone.0155818 27191859 PMC4871481

[57] Narula MK Ahuja GK Whig J Narang AP Soni RK. Status of lipid peroxidation and plasma iron level in bronchial asthmatic patients. Indian J Physiol Pharmacol (2007) 51(3):289–92. 18341227

[58] Urushidate S Matsuzaka M Okubo N Iwasaki H Hasebe T Tsuya R. Association between concentration of trace elements in serum and bronchial asthma among Japanese general population. J Trace elements Med Biol (2010) 24(4):236–42. doi: 10.1016/j.jtemb.2010.06.001 20832272

