# Supplementary material for: Relationship between serum iron and blood eosinophil counts in asthmatic adults: data from NHANES 2011-2018
Source: Front Immunol. 2023 Sep 4;14:1201160. doi: 10.3389/fimmu.2023.1201160 (PMC10507334; doi:10.3389/fimmu.2023.1201160)
Supplement: Supplementary file 1 [file DataSheet_1.docx]

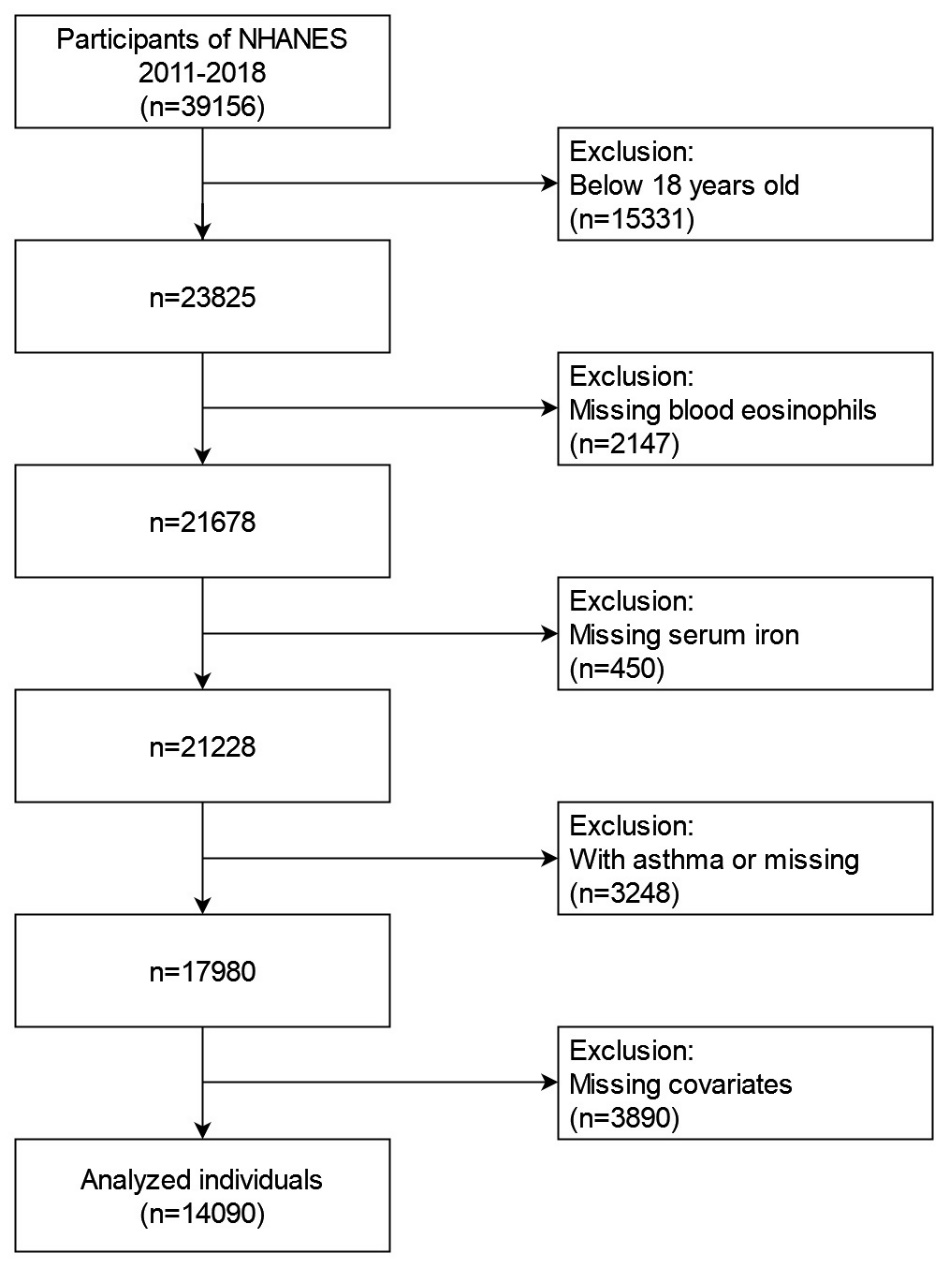


**Supplementary figure 1.** Flowchart for choosing non-asthmatic subjects.

| Supplementary table 1. Three weighted linear regression models explicate the link of the serum iron with blood eosinophils counts among adults without asthma. | | | |
| --- | --- | --- | --- |
|  | Model 1 | Model 2 | Model 3 |
|  | β (95% CI) P value | β (95% CI) P value | β (95% CI) P value |
| Serum iron | -1.05 (-1.58, -0.52) 0.0003 | -1.65 (-2.22, -1.09) <0.0001 | -1.16 (-1.71, -0.61) 0.0002 |
| Serum iron quartile |  |  |  |
| Q1(0.40-10.89) | Reference | Reference | Reference |
| Q2(10.90-14.49) | -6.58 (-15.31, 2.15) 0.1447 | -15.46 (-24.51, -6.42) 0.0015 | -10.74 (-19.81, -1.67) 0.0261 |
| Q3(14.50-18.79) | -11.94 (-21.63, -2.24) 0.0190 | -23.67 (-33.64, -13.70) <0.0001 | -16.05 (-25.64, -6.47) 0.0023 |
| Q4(18.80-85.30) | -17.50 (-28.30, -6.71) 0.0024 | -30.05 (-41.38, -18.71) <0.0001 | -20.41 (-31.67, -9.15) 0.0011 |
| P for trend | 0.0013 | <0.0001 | 0.0007 |
| Note: Model 1 adjusted no covariates. Model 2 adjusted sex, age and race. Model 3 adjusted sex, age, race, educational background, marital state, poverty to income ratio, body mass index, smoking state, alcohol intake, vitamin A intake, vitamin B12 intake, vitamin C intake, folate intake, iron intake, hypertension history, diabetes history and steroid drugs use. Q1-Q4: Serum iron are grouped by quartile. | | | |

| Supplementary table 2. Stratified correlation of serum iron with blood eosinophil counts in adults without asthma. | | | |
| --- | --- | --- | --- |
| Subgroup | N | β (95% CI) P value | P-interaction |
| Sex |  |  | 0.613 |
| Male | 7046 | -1.02 (-1.84, -0.19) 0.0204 |  |
| Female | 7044 | -1.31 (-2.04, -0.57) 0.0013 |  |
| Age |  |  | 0.9386 |
| <40 | 4696 | -1.13 (-1.73, -0.52) 0.0009 |  |
| 40-60 | 4732 | -1.09 (-2.18, -0.00) 0.0577 |  |
| >=60 | 4662 | -1.36 (-2.69, -0.04) 0.0518 |  |
| Race |  |  | 0.577 |
| Mexican American | 1971 | -1.39 (-2.53, -0.24) 0.0236 |  |
| Other Hispanic | 1412 | -0.58 (-2.70, 1.54) 0.5960 |  |
| Non-Hispanic White | 5564 | -1.06 (-1.87, -0.24) 0.0162 |  |
| Non-Hispanic Black | 2940 | -2.04 (-3.02, -1.05) 0.0003 |  |
| Other Race | 2203 | -1.26 (-2.34, -0.19) 0.0271 |  |
| Education |  |  | 0.9893 |
| Less than high school | 2860 | -1.10 (-2.41, 0.21) 0.1092 |  |
| High school | 3202 | -1.10 (-2.37, 0.17) 0.0973 |  |
| More than high school | 8028 | -1.19 (-1.88, -0.50) 0.0018 |  |
| Marital status |  |  | 0.9208 |
| Married | 7326 | -1.16 (-1.98, -0.34) 0.0086 |  |
| Single | 5557 | -1.11 (-1.89, -0.33) 0.0084 |  |
| Living with a partner | 1207 | -1.40 (-2.69, -0.10) 0.0416 |  |
| Poverty to income ratio |  |  | 0.7623 |
| Low | 4670 | -0.89 (-1.83, 0.06) 0.0744 |  |
| Middle | 4714 | -1.03 (-2.01, -0.05) 0.0461 |  |
| High | 4706 | -1.40 (-2.30, -0.50) 0.0042 |  |
| BMI |  |  | 0.2161 |
| <25 | 4077 | -0.68 (-1.71, 0.34) 0.2001 |  |
| 25-28 | 2853 | -1.98 (-2.89, -1.07) 0.0001 |  |
| >=28 | 7160 | -1.33 (-2.33, -0.34) 0.0127 |  |
| Smoked at least 100 cigarettes in life |  |  | 0.2453 |
| Yes | 5987 | -1.48 (-2.24, -0.71) 0.0005 |  |
| No | 8103 | -0.88 (-1.62, -0.14) 0.0251 |  |
| Hypertension |  |  | 0.5919 |
| Yes | 5025 | -0.85 (-2.15, 0.44) 0.2039 |  |
| No | 9065 | -1.28 (-1.95, -0.61) 0.0007 |  |
| Diabetes |  |  | 0.4093 |
| Yes | 1864 | -0.43 (-3.49, 2.62) 0.7819 |  |
| No | 11868 | -1.26 (-1.80, -0.72) 0.0001 |  |
| Borderline | 358 | 0.90 (-2.41, 4.21) 0.5977 |  |
| Steroid drugs use |  |  | 0.9951 |
| Yes | 536 | -1.17 (-5.40, 3.05) 0.5895 |  |
| No | 13554 | -1.16 (-1.73, -0.60) 0.0003 |  |
| Note: Above adjusted sex, age, race, educational background, marital state, poverty to income ratio, body mass index, smoking state, alcohol intake, vitamin A intake, vitamin B12 intake, vitamin C intake, folate intake, iron intake, hypertension history, diabetes history, and steroid drugs use. The model was not adjusted for the stratification variable in any of the cases. | | | |
